# Pain Assessment and Management in Pediatric Trauma Patients Transported to an Emergency Department: A Retrospective Cohort Study

**DOI:** 10.3390/children13050593

**Published:** 2026-04-24

**Authors:** Kaja Kubiak, Tomasz Konieczny, Mateusz Henryk Kopczyński, Jonasz Jurek, Natalia Wierzejska, Aneta Michalczewska, Joanna Żyła, Jan Stachurski

**Affiliations:** 1 Student Research Association of Pediatric Emergency Medicine, Medical University of Warsaw, 02-091 Warsaw, Polandaneta.michalczewska@wp.pl (A.M.);; 2 Zakład Ratownictwa Medycznego WUM, Department of Emergency Medical Services, Faculty of Health Sciences, Medical University of Warsaw, 02-091 Warsaw, Poland

**Keywords:** pediatric emergency medicine, emergency treatment, pain measurement, pain management, acute pain, analgesics

## Abstract

**Highlights:**

**What are the main findings?**

- Despite documentation of pain in 85.5% of pediatric trauma patients, only 21.2% received analgesics, and at least 70.2% of the cohort had no documented evidence of treatment fulfilling Polish Ministry of Health recommendations for prehospital pediatric pain management.
- Combined opioid regimens (fentanyl with metamizole, morphine or paracetamol) were the most effective in achieving the recommended ≥50% pain reduction; however, pain reassessment was frequently performed prematurely—in some cases within one minute of drug administration—rendering efficacy evaluation unreliable.

**What are the implications of the main findings?**

- National prehospital pediatric pain guidelines should be updated to include paracetamol as a recommended option for mild pain, and to specify a minimum time interval before pain reassessment to ensure clinically meaningful evaluation of analgesic effectiveness.
- Systemic interventions—particularly the introduction of a unified electronic EMS documentation system and the Ministry of Health recommendations in 2019—were associated with improved rates of pain documentation, demonstrating that regulatory and technological changes can meaningfully drive quality of care in prehospital pediatric emergency medicine.

**Abstract:**

Objectives: To evaluate how often pain is assessed and treated in pediatric trauma patients transported by Emergency Medical Services (EMS) to a pediatric emergency department (ED), and to compare current practice with national recommendations of the Polish Ministry of Health for prehospital pediatric pain management. Methods: We conducted a retrospective analysis of EMS and ED documentation for all trauma patients under 18 years of age transported to the Pediatric Teaching Hospital of the University Clinical Center of the Medical University of Warsaw between 1 January and 31 December 2021. A total of 981 patients with injury or suspected injury or burns were included without exclusion criteria. For patients with documented pain scores, we analyzed pain intensity (0–10), the scales used [Visual Analog Scale (VAS), Numerical Rating Scale (NRS), Wong–Baker Faces Pain Rating Scale (FACES)], body region injured, Glasgow Coma Scale (GCS) score, suspected alcohol or psychoactive substance use, and type and route of analgesic administration. We further evaluated non-pharmacological interventions, pain reassessment, and achievement of at least 50% pain reduction, as defined in national guidelines. Statistical analysis included Student’s *t*-test or ANOVA for quantitative variables and maximum likelihood chi-square tests for qualitative variables (α = 0.05). Results: Pain was assessed in 839/981 (85.5%) patients; 651/839 (77.6%) reported pain, most frequently of moderate intensity. Despite this, only 208/981 (21.2%) patients received analgesics prehospitally. Morphine and paracetamol were the most frequently used drugs, predominantly administered intravenously, while non-opioid monotherapy was commonly used in patients with lower baseline pain scores. Less than half of all patients received any non-pharmacological intervention whatsoever. Pain was reassessed in 734/839 (87.5%) patients, with a mean reassessment time of approximately 10 min; however, in many cases reassessment occurred earlier than the expected onset of analgesic action. Overall, only 29.4% of patients with pain and documented reassessment achieved the recommended ≥50% reduction in pain intensity, and at least 70.2% of the cohort had no documented evidence of treatment fully complying with national recommendations. Conclusions: In this real-world prehospital and ED cohort, pediatric trauma pain remains under-treated, and adherence to national guidelines on opioid-based analgesia and pain reassessment is suboptimal. Further efforts are needed to improve documentation, expand the recommended pharmacological options for mild pain, and strengthen education on guideline-concordant pediatric pain management in EMS.

## 1. Introduction

Pain is one of the most common reasons for Emergency Department (ED) visits in both adults and children, with more than 40% of adolescents reporting pain during their ED stay [1,2,3,4,5]. Unfortunately, it is often underestimated and left untreated [3,6,7]. Simultaneously, prehospital pain evaluation is often omitted from documentation [1,5]. Not every trauma patient receives pain treatment; rarely in the correct dose [1,7,8]. When compared, children report higher pain scores than estimation made by medical staff [9,10,11]. Some studies indicate that parents also tend to underestimate their child’s pain [11,12]. Therefore, it is important to use appropriate pain scales to assess pain perception as accurately as possible. Furthermore, pain intensity of pediatric patients is estimated and documented dramatically less often than adults’ [13]. Using appropriate pain scales and recording pain scores is important, as it increases the likelihood of administering pain relief to trauma patients and reduces the wait time for administration of analgesics [14,15]. It is therefore crucial, because without appropriate pain assessment children and adolescents receive less pain relievers compared to adults [13].

Adequate pain management is especially important in prehospital care. Studies show that patients treated by Emergency Medical Services (EMS) receive analgesia faster than patients treated in the ED, and the actual analgesics delivery time in the ED is longer than expected [16,17]. Many studies show that acute treatment with morphine can reduce the severity of post-traumatic stress disorder (PTSD) symptoms in injured children [18,19,20,21]. At the same time, rapid and adequate pain relief is thought to be one of the most important factors influencing parent satisfaction [22].

Our study aimed to establish how often current recommendations of the Polish Ministry of Health are followed in daily practice and to determine the need for new practical guidelines to ensure adequate pain validation and effective pain relief in the ambulance before admitting the patient to the ED. The recommendations are presented in Table 1.

## 2. Materials and Methods

We conducted a retrospective analysis of the medical documentation of patients transported by the Emergency Medical Service (EMS) to the Emergency Department (ED) of the Pediatric Teaching Hospital in Warsaw, Poland. Data for the study were collected from 1 January 2021 to 31 December 2021. We selected 981 patients who met inclusion criteria: age under 18 years, injury or suspected injury or burns. The exclusion criteria included a lack of complete documentation, patients transferred from another hospital or patients self-admitted to an emergency department. The database was constructed based on the acquired data, which were: age, sex, localization, character, and mechanism of trauma, suspicion of alcohol or psychoactive substance abuse, suspicion of suicide, vital signs, Glasgow Coma Scale (GCS) score, mean pain score, pain reassessment score and time, administered treatment, and procedures.

Self-reported pain intensity was assessed using various pain scales: Visual Analog Scale (VAS), Numerical Rating Scale (NRS), and Wong–Baker Faces Pain Rating Score (FACE). Non-numerical scales scores were converted to a numerical pain rating scale from 0 to 10. VAS score in millimeters was divided by 10 and later rounded to the nearest integer. FACE scale was converted to numbers using the official tool—achievable pain scores were 0, 2, 4, 6, 8 and 10 as the correlations of these scales have been reported in the literature [23,24]. Based on these scales, we categorized pain intensity into three grades: mild (1–3), moderate (4–6), and severe (7–10) [25]. A reported pain score of 0 indicated the absence of pain.

We conducted an analysis of pain intensity of patients in relation to the injury of different specific body parts. Additionally, we compared groups of patients presenting with isolated injury of only one part of the body. Furthermore, to assess whether depressed consciousness affects pain perception, we divided patients according to the Glasgow Coma Scale (GCS) into 3 groups: mild (14–15), moderate (9–13), and severe (3–8) [26].

Patients who received analgesics were analyzed in terms of sex, age, type, time, and route of analgesic used, as well as their effectiveness: the reduction in pain intensity after drug administration. Assessment of effectiveness of used analgesics was analyzed depending on whether they were administered as monotherapy or in combination with other analgesics.

Based on the recommendations of the Polish Ministry of Health (PMOH) “Good Practices in Pain Treatment for Children in Emergency Medical Services”, we establish the cut-off point for satisfactory treatment efficiency as a 50% reduction in reported pain [27]. Moreover, to evaluate reduction in pain intensity after the applied treatment, we used the mean pain scores and aforementioned pain grades.

The analysis was conducted using STATISTICA 13 software. Differences between quantitative variables were tested using Student’s *t*-test or ANOVA test, and qualitative variables were tested using the Chi-square test. The significance level was set at 0.05.

The data used in this study were provided by the Emergency Department of the Pediatric Teaching Hospital, University Clinical Centre of the Medical University of Warsaw, Poland. Authorization for access to patient documentation and publication was approved by the hospital director. Ethical approval for our study was obtained from the Ethics Committee of the Medical University of Warsaw with decision number AKBE/80/2022. According to the Helsinki Declaration, all data were fully anonymized, preventing any possibility to identify the patients.

## 3. Results

**Figure 1:**
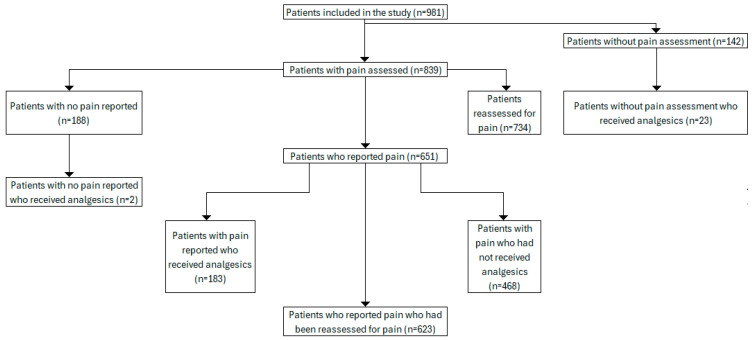
Flow chart of patient group analyzed in the study.

In 2021, there were 981 pediatric trauma patients transported by Emergency Medical Services to the Emergency Department of the Pediatric Teaching Hospital in Warsaw. The mean age of the participants was 8.32 (SD = 5.74). Enrolled patients were between 9 days and 17 years 8 months old. The majority were male (569/981, 58%). The distribution of the patients in particular groups which was used to analysis is presented in Figure 1.

Among the patients analyzed, not everyone had their pain assessed. Out of 981 patients, we included only those who had their pain assessed using one of the pain scales in the pain measurement analysis, resulting in only 839 (839/981, 85.52%) patients.

Among the 839 patients with documented pain scores, 188 (22.41%) reported no pain. Out of those who reported pain, moderate pain was the most frequent (288/839, 34.33%). The least common was severe pain (199/839, 14.18%). Detailed pain scores reported by analyzed patients are presented in Table 2.

In total, 487 male and 352 female patients had their pain intensity estimated. The mean pain score of boys was 3.56 (SD = 2.76) and was significantly higher than the mean pain score of girls 3.16 (SD = 2.74) (*p* = 0.04).

### 3.1. Pain Scales

**Figure 2:**
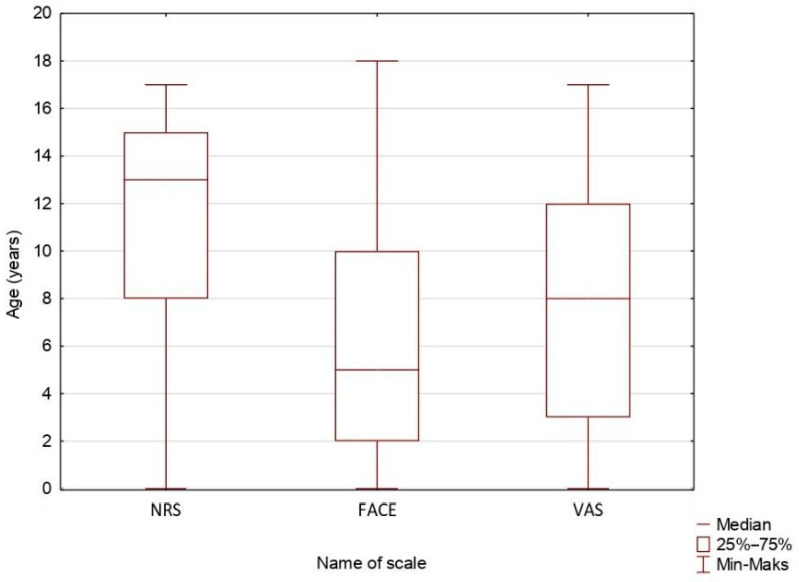
Distribution of scales according to age.

The mean age of the participants was 8.32 (SD = 5.74). The Numerical Rating Scale (NRS), which was used by 361 (361/839, 43.03%) children, was on average most frequently used in older patients. The mean age of children in whom the NRS was used was 11.35 (SD = 5.12), which was higher than the mean age of children assessed using other scales (*p* < 0.01). The mean age of the children assessed with Visual Analog Scale (VAS) was 7.64, (SD = 4.94) (58 patients (58/839, 6.91%)) and the mean age of those assessed with the Wong–Baker Faces Pain Rating Score (FACE) was only 6.20 (SD = 5.03) (397 patients (397/839, 47.32%). The distribution of the scales depending on the age is presented in Figure 2.

Mean pain scores of all pain scales differ significantly (*p* < 0.01). The mean score of patients assessed with NRS (mean score = 3.02) was lower than the mean score of patients assessed with FACE (mean score = 3.71) (*p* < 0.01). Differences in pain scores between other scales were not significant. The mean VAS score was 3.47.

### 3.2. Pain Intensity and Injured Part of the Body

According to the used scales, patients with head injuries (591 patients) experienced lower pain intensity than patients with injuries in the other locations (*p* < 0.01). The mean pain score of patients with head injuries was 2.90, compared to 4.10 of patients without head injuries. We divided patients with head injuries into three groups according to the Glasgow Coma Scale (GCS). Out of 591 children with head injuries, only 443 had their GCS assessed. The mean pain scores reported by these groups are presented in Table 3. Among patients with minor GCS, those with head injuries had a significantly lower mean pain score (2.85, SD = 2.46) compared to patients who suffered from the other types of injuries (mean 4.13, SD = 2.97) (*p* < 0.01).

Injuries to other parts of the body were associated with an increase in pain report. We compared patients with injuries to a specific body part with patients without those specific injuries, but with injuries to other body parts. The intensity of pain experienced by patients with neck injuries (37 patients) was significantly higher than in patients without these injuries (*p* < 0.01). The mean pain score in patients with neck injuries was 4.97, compared to 3.33 in those with other injuries that were not associated with a neck injury. Moreover, children with chest injuries (76 patients) reported higher intensity of pain (mean 4.79) than patients without these injuries (mean 3.27) (*p* < 0.01). Similarly, children with abdominal and pelvic injuries (60 patients) demonstrated a higher mean pain score (4.67, SD = 2.61) compared to those without such injuries (mean 3.31, SD = 2.74) (*p* < 0.01). Patients with upper limb injuries (228 patients) resulted in a higher mean pain score (4.19) compared to patients without these injuries (mean 3.14) (*p* < 0.01). Similarly, the mean pain score in children with lower limb injuries (177 patients) was 4.43, while in patients without this injury it was 3.15 (*p* < 0.01). Pain intensity data for injuries to specific body parts are presented in Table 4.

### 3.3. Isolated Injuries

Furthermore, we selected all injured patients with isolated injuries and divided them into groups according to the injured part of the body. The study demonstrated that isolated pain of head is less painful than isolated pain of upper (*p* < 0.01) or lower limb (*p* < 0.01). The mean pain score of patients with isolated head injuries was 2.54, while for isolated upper limb injury it was 4.16 and lower limb 5.05. The other isolated injuries did not correlate with reported pain intensity. The data regarding the intensity of reported pain, according to the location of the injuries, are presented in Table 5.

### 3.4. Depressed Consciousness

The analysis confirmed that level of consciousness did not correlate with the assessment of pain (*p* = 0.78). To establish this, we compared patients with GCS 15 (751) and those with GCS less than 15 (25). In total, 63 patients did not have their GCS assessed.

Children suspected of alcohol abuse (20) experienced milder trauma-related pain (mean 1.60) than the sober ones (mean 3.44) (*p* < 0.01). The same correlation was observed in patients suspected of being under the influence of psychoactive substances (*p* = 0.01). These patients (4) did not declare any pain (mean 0.00).

### 3.5. Pain Treatment and Reassessment

Pain management analysis was conducted on a larger group of all 981 patients transported to the ED by EMS, because not every patient who received analgesic or non-pharmacological treatment had their pain assessed. Of all 981 patients transported to the ED, 208 (208/981, 21.2%) received pain relievers. Of these, 137 (137/569, 24.08%) were boys and 71 (71/412, 17.23%) were girls. There were 23 (23/981, 2.34%) patients who received analgesics without pain assessment. Two patients received analgesic treatment with a pain score of “0”. Out of 651 patients who reported pain, there were 468 (468/651, 71.89%) patients who did not receive any pain relivers. Additional data regarding the number of treated and untreated patients in terms of their pain assessment are presented in Table 6.

The group of children who received antinociceptive drugs was significantly older, reaching a mean age of 11.06 years, compared to those who did not with a mean age of 7.86 years (*p* < 0.01). On admission, boys scored on average 0.4 points higher on pain scales than girls (3.56 vs. 3.16) (*p* = 0.04). Boys received fentanyl almost twice as often (45/569, 7.91% vs. 18/412, 4.37%) (*p* = 0.01).

The mean pretreatment pain score of children receiving analgesics was 6.64. Patients who did not receive analgesics had a mean pain score of 2.5. Antinociceptive drugs were administered to 208 (208/981, 21.2%) patients. In total, 180 (180/208, 86.54%) of them received drugs intravenously, while 7 (7/208, 3.36%) of them were treated intranasally, 5 (5/208, 2.4%) per os, and 4 (4/208, 1.92%) per rectum. None of the routes of administration correlated with a decrease in pain score after drug administration. The most commonly used drugs were morphine—administered in 74 cases (74/208, 35.58%) and paracetamol—administered 73 times (73/208, 35.1%). Both were mostly used alone. The frequency of analgesic administration, pain scores reported by patients prior to drug administration, and mean pain reduction after reassessment are presented in Table 7. Fentanyl was administered to 63 patients (63/208, 30.29%), metamizole to 21 (21/208, 10.1%), and NSAIDs to 11 (11/208, 5.29%). Fentanyl and morphine (in monotherapy or with other drugs) were mostly commonly used for severe pain (*p* < 0.01), reaching a mean of 7.4 (SD = 2.13) for fentanyl and 7.38 (SD = 1.52) for morphine. Metamizole, paracetamol, and NSAIDs were used in the event of relatively weaker pains. Paracetamol was used in 50% of cases for moderate pain and 42.65% for severe pain (*p* < 0.01), while metamizole was used in 45% of cases for both moderate and severe pain (*p* < 0.01).

Of all 981 patients transported to ED by EMS, 416 (416/981, 42.41%) received non-pharmacological treatment. Distribution of non-pharmacological treatments was presented in Table 8. Out of all 208 treated patients, only 135 (135/208, 64.90%) received both—analgesics and non-pharmacological treatment.

Of the 839 patients whose pain was assessed, it was reassessed in only 734 (734/839, 87.49%) cases. Mean time to reassess pain was 9 min 53 s. The longest time to reevaluation was 77 min and the shortest—5 s. In total, 111 patients were reassessed in less than 2 min, while 15 of them in less than 1 min. Specific reassessment times are presented in Table 9.

Of the 623 children who experienced any trauma-related pain and had their pain reassessed, 231 felt mild pain. In reassessment, 215 (215/231, 93.07%) still experienced mild pain and 16 (16/231, 6.93%) of them had no pain. A total of 279 patients suffered from moderate pain. None of them progressed to severe pain. Moreover, 177 (177/279, 63.44%) still experienced moderate pain, but 92 (92/279, 32.97%) felt only mild pain in reassessment and 10 (10/279, 3.85%) reported no pain. There were 113 patients with severe pain. On reevaluation, 8 (8/113, 7.08%) of them experienced no pain, 36 (36/113, 31.86%) had mild pain, 53 (53/113, 46.9%) moderate. In total, 16 (16/113, 14.16%) patients remained in severe pain. Generally, 50% pain reduction at the time of reassessment was achieved in 183 cases (183/623, 29.37%). No treatment was needed to reduce pain in 42.08% of these cases. However, they constituted only 17.15% of the untreated group. Using analgesics was more efficient than not using any treatment to achieve 50% pain reduction, as it was obtained in 60.92% of analgesic treatment cases (*p* < 0.00). Moreover, 50% pain reduction was significantly more common in the severe pain group, in which it was observed in 61.06% cases. For the moderate pain group, 30.82% achieved the goal, whereas in the mild pain group, it was noted in only 12.12% cases.

Interestingly, pain management of isolated head injuries was significantly less efficient than isolated lower and upper extremity injuries. In head trauma, analgesics reduced pain on average by 0.49, in upper extremity trauma by 1.8 (*p* < 0.01) and in lower extremity trauma by 2.28 (*p* < 0.01).

Treatment with fentanyl with metamizole or morphine or paracetamol was the most effective, reaching a mean pain score reduction of over 5. Morphine with paracetamol, fentanyl alone, and morphine alone achieved mean pain reductions of around 4 points. Paracetamol with metamizole, paracetamol alone, metamizole alone, and NSAIDs alone were much less effective. Their pain reduction ranged from 1.57 to 2.6. Patients without analgesics experienced only a 0.41 pain reduction.

Treatment with paracetamol (*p* < 0.01), morphine (*p* < 0.01), morphine with paracetamol (*p* < 0.01), fentanyl (*p* < 0.00), fentanyl with metamizole (*p* < 0.01), and fentanyl with morphine (*p* < 0.01) were statistically more efficient in reducing pain than no treatment. When fentanyl was administered together with metamizole, it decreased pain more efficiently by 4.06 than paracetamol with metamizole (*p* = 0.02), paracetamol alone by 4.58 (*p* < 0.01), metamizole alone by 4.78 (*p* < 0.01), and NSAIDs alone by 5.09 (*p* < 0.01). At the same time fentanyl with morphine worked better than NSAIDs by 3.68 (*p* = 0.01) and metamizole by 3.37 (*p* = 0.04). Combining fentanyl with paracetamol decreased mean pain score better than NSAIDs by 3.63 (*p* < 0.01), metamizole by 3.32 (*p* = 0.01), and paracetamol alone by 3.12 (*p* = 0.02). Fentanyl, when used alone, was found to be superior to NSAIDs in pain relief by 2.7 (*p* = 0.02), metamizole by 2.41 (*p* = 0.03), and paracetamol by 2.21 (*p* = 0.00). Morphine alone was found to manage pain better than NSAIDs by 2.8 (*p* = 0.01), metamizole by 2.49 (*p* = 0.02), and paracetamol by 2.29 (*p* < 0.01).

## 4. Discussion

Pain is a prevalent symptom that frequently occurs after body injuries and should be expected by medical providers in the context of trauma [18]. An appropriate approach to assessment and management has been shown to improve clinical outcomes, and neglect can have lifelong psychological consequences [6,28,29,30].

### 4.1. Assessment of Pain

Pain assessment should precede administering analgesia to provide information to assess improvement in reducing pain experienced by patients. Pain assessment was documented by the EMS in only 85.52% of the enrolled patients, despite the Ministry of Health (MoH) Pain Management Recommendation for EMS requiring pain assessment to be performed in every patient. We did not identify objective reasons for the lack of documented pain assessment in these patients. While some of the patients included in our study had their pain intensity not assessed, there is nevertheless notable improvement over the data reported by Holak et al. Similarly, Holak et al. analyzed a similar group of patients also transported by EMS in an area around Warsaw in 2017 and 2018. They also used EMS documentation as a data source and had a similar sample size (981 to 1604 injured patients), considering that our study did not analyze patients who received care by EMS and were not subsequently transported to a hospital, who were 30% of another study cohort (0.82% of patients had their pain assessed in this study) [7]. Although it cannot be confirmed without cross-reviewing EMS documentation with another source, an improvement in documentation quality may have been a partial reason for this improvement, which may be related to the introduction of a pain assessment tool in the EMS electronic management system in December 2019 [31]. Additional changes such as the introduction of Polish Ministry of Health (PMOH) recommendations in 2019, and legislation changes in 2017 may have impacted EMS practices in pain management [27,32]. Also, 14.48% patients unassessed for pain was similar to 18.8% reported by Lord et al. and significantly better than 25% and 32% reported by Brown et al. and Murphy et al., respectively [5,33,34].

We identified sex differences, especially in the higher mean pain scores observed in the male pediatric patient group, which match findings of some studies, but we were unable to confirm if the higher pain scores reported in boys were justified or not, and if any sex has a tendency to be assessed with a higher pain score when presented with a similar condition [35]. However, higher pain ratings given to boys may have reflected assessor bias in the perceived intensity of pain. In this study, boys were consistently rated as experiencing higher pain than girls, despite identical clinical conditions and identical pain-related behaviors across all study groups [35]. While many of studies using experimental methods reported no difference in pain assessment due to sex of child or adolescent, meta-analysis performed by Boerner et al. showed a significant difference with females reporting higher pain intensity for the same stimuli, which is opposite to our study. However, without additional analysis correlating injury severity, its effect on the intensity of nociceptive stimuli with reported pain, we are unable to support or deny this claim [36,37,38,39]. The lower average age of patients assessed with FACE or VAS compared to NRS was adequate with intended use of these scales in younger patients. The MoH recommendation does not specify any specific age criteria of pain scale choice [40]. While there are small significant differences in pain scores with the use of different scales, without additional data and analysis of injury severity, we cannot rule if the difference is caused by differences in groups of patients or differences in scales. While small differences between FACE, VAS and NRS were shown in the literature, they do not seem to interfere with their intended clinical use [41,42,43]. We found no instances of documented use of behavioral pain assessment scales in our study, which may have indicated that the youngest patients may not receive appropriate pain management, which is a problem reported by Holak et al. [7].

### 4.2. Management of Pain and Reassessment

While the pain occurrence was found in 66.36% of our analyzed cohort, not all of them were subjected to procedures intended for pain management. In total, 21.2% of patients received analgesics, which was an improvement over the data reported by Holak et al. (16%) [7]. In both cases, this is a deviation from practices included in recommendations. While using fentanyl and morphine in pediatric trauma patients is a recommended approach by some authors and the Polish recommendation, 76 out of 208 patients who received treatment were administered only non-opioid medications [44]. Although only non-opioid medication resulted in lower pain reduction (2.1 to 4.36 in the group that received opioids), it was mostly utilized in patients presented with lower pain intensity (5.29 to 7.37 respectively), resulting in a similar average final pain intensity score (3.19 to 3.01). These results, with popularity and safety of paracetamol, which was the most used medication in our patients after opioids, can be used in the consideration about placing it in Polish recommendation, especially in patients with mild pain, with a note that other authors do not recommend use of opioids in pediatric trauma patients with mild pain [44]. Although in our study we reported higher rate of documented pain assessment than some authors, it did not result in higher medication rate, while Murphy et al. reported 26% patients receiving analgesic medication and Lord et al. reported higher 39.5% (with similar assessment rate) [5,34]. With data presented by Holak et al., with documented assessment lower than 1% and 16% treatment rate, we observed a lack of simple correlation between documented assessment of pain and the administration of medication. Further, Jaeger et al. also reported no change in the proportion of patients treated with analgesia after a significant increase in the documentation of pain scores [45]. While the use of non-pharmacological methods, immobilization, cervical collars, spinal boards and dressings (either alone or in combination), was documented in 208 patients, we found no instances of documented cooling usage. Despite inclusion of cooling in the Polish national recommendation, the legislation does not require ambulances to be equipped with the appropriate resources [27,46].

While the reassessment rate (87.49%) was significant, the relevance of many reassessments is questionable due to the short time frame between the administration of analgesic and reassessment, which in many cases was less than the time of action onset of the used medication [47,48,49]. Additional efforts may be required by healthcare regulators to improve EMS personnel awareness of the importance of pain reassessment in the pain management process [44].

### 4.3. Adherence to Polish Guidelines

The results of our study showed that at least 70.23% of pediatric patients transported to ED had no documented evidence of treatment fulfilling recommendations of the PMOH “Good Practices in Pain Treatment for Children in Emergency Medical Services”. Of these patients, 142 had no pain assessment, 468 experienced pain but did not receive any administration of analgesic, 76 were treated only with non-opioid medications and 3 patients received opioids but did not have their pain intensity reassessed.

The limitation of the choice of therapeutic agent in pediatric trauma patients to fentanyl and morphine (with ketamine available only for EMS crews consisting of a Medical Doctor (MD), which are the minority of EMS crews in Poland, with 25.78% of prehospital interventions in a similar patient group to our study of EMS performed by them in data presented by Holak et al.), may be disputed with 29.08% of patients who reported mild pain and EBM-based recommendations not supporting the usage of opioids in patients with pain which is neither moderate nor severe [7,44]. The use of acetaminophen, which is already a common treatment provided by EMS, may be proposed as it is already included in guidelines for acute pain management published by the Polish Society of Anesthesiology and Intensive Care, and its safety and efficacy in pediatric population is well established [50,51,52]. Introduction of this medication in recommendations can help alleviate providers’ hesitance to administer analgesic in patients with a low level of pain. Adherence to requirement of assessing pain scores has improved after implementation of MoH recommendations in late 2019 [7]. Additional emphasis should be put on the reassessment of pain due to problems highlighted in our study, specifically the lack of performance and inadequate time delay.

### 4.4. Limitations

Due to the limitations of the patient samples and the duration of the study, the data may not be representative of smaller patient groups, especially those with severe trauma, multi-organ trauma and after traumatic cardiac arrest. Another possible issue that may impact our results is the quality of the documentation that was used as the source of data. As shown in the results of our data, a significant part of EMS documentation was performed without taking into account the onset of action times. Occurrence of reassessment time shorter than the onset of action times of used analgesics may obscure the true efficacy of the treatment administered. Additionally, patients who experience pain in an atypical way due to neurological or psychological reasons might have been misinterpreted. In the present study, we limited our analysis to patients who were transported to a single, specific Emergency Department. Consequently, our findings may not be fully applicable to the wider population. Due to pooling of different pain scales for part of the statistical analysis, some additional variables may not be identified.

## 5. Conclusions

Our findings suggest that pain assessment and management in trauma patients is often undervalued by Emergency Medical Services. We found that in 2021, a substantial proportion of pediatric trauma patients transported to the Emergency Department by EMS did not have their pain formally assessed.

There is a need to pay more attention to analgesic treatment among children. The frequency of administering pain relief, particularly guideline-recommended regimens, was disproportionately low compared with the number of children reporting pain. Most trauma patients reported pain, but only about a quarter of them received analgesic medications. According to recommendations of the Polish Ministry of Health (PMOH) and EMS documentation, only 14.59% trauma patients who reported pain received appropriate treatment, including fentanyl or morphine in monotherapy with non-pharmacological interventions [27]. Absence of usage of cooling may be in part attributed to omittance of documenting it. In our study, the most used analgesics in monotherapy were morphine and paracetamol, and their combination was the most frequent.

The choice of antinociceptive depended on pain intensity. Patients who reported weaker pain were often treated with metamizole, paracetamol and NSAIDs, contrary to the severe pain patients’ group who received fentanyl and morphine. Most analgesics were administered intravenously, as recommended. Adhering to recommendations, only a small part of the patients received medications intranasally, per os or per rectum. However, against PMOH guidelines, less than two-thirds of pharmacologically treated patients received non-pharmacological interventions.

We emphasize the need for accurate pain reassessment by EMS. Unfortunately, not every patient undergoing treatment had the effectiveness of introduced treatment evaluated by reassessing the pain intensity after administration of analgesics. Furthermore, pain intensity revaluation was performed too early, sometimes in less than one minute. In future updates to guidelines, sufficient time before reassessing pain should be included as necessary practice to ensure high quality of care. Undoubtedly, accurate pain revaluation is crucial, because reassessing pain intensity too quickly may result in an inaccurate estimation of treatment effectiveness due to the longer onset time of analgesics.

It is important to highlight that as a result of the administered treatment, patients reported lower or the same intensity level of pain than before the treatment. However, in most cases the pain reduction did not meet the PMOH criterion [27]. The recommended 50% pain score reduction was noted in less than one-third of treated patients. Interestingly, almost half of the patients who reached the required purpose did not receive any pharmacological treatment.

The effects of introduction of unitary electronic documentation system and pain treatment guidelines in EMS on improvement of quality of care can be used as a positive example in developing prehospital medical care.

Studies featuring parallel applications of different pain scales or randomized usage of these scales in a pediatric trauma setting should be performed in future aiming to deliver more definitive data about the existence of a difference between results delivered by usage of different scales.

Further developments in assessment and treatment should be made. There is still a lot of work to be done with the creation of new appropriate and more detailed guidelines, which will be commonly used in daily practice and ensure satisfactory pain reduction.

## Figures and Tables

**Table 1:** Current (full) recommendations of the Polish Ministry of Health regarding treatment of pediatric trauma patients.

Treatment	Drug Administration	Target of Treatment
Morphine or Fentanyl or Ketamine (children above 15 y.o) + non-pharmacological treatment	Intravenous or Intraosseous	50% reduction in pain intensity

**Table 2:** Pain distribution of assessed patients.

Pain Score	Number of Patients	Grade of Pain
0	188 (22.41%)	None 188 (22.41%)
1	33 (3.93%)	Mild 244 (29.08%)
2	150 (17.88%)	
3	61 (7.27%)	
4	174 (20.74%)	Moderate 288 (34.33%)
5	39 (4.65%)	
6	75 (8.94%)	
7	19 (2.26%)	Severe 119 (14.18%)
8	68 (8.10%)	
9	6 (0.72%)	
10	26 (3.10%)	
TOTAL	839	839

**Table 3:** Mean pain scores according to grades of GCS score.

GCS Score	Number of Patients with Head Injury	Mean Pain Score	Standard Deviation
minor (13–15)	430	2.85	2.46
moderate (9–12)	8	2.38	2.13
severe (3–8)	5	3.4	1.67
Total	443		

**Table 4:** Mean pain scores of patients according to area of injury and *p*-value of their differences.

Injured Area	Number of Injured Patients	Mean Pain Score of Injured Patients	Number of Patients with Injuries in Other Location	Mean Pain Score of Patients with Injuries in Other Location	*p*-Value of Differences in Mean Pain Scores Between Comparison Groups
Head	591 (70.44%)	2.90	248 (29.56%)	4.10	0.000000
Neck	37 (4.41%)	4.97	802 (95.59%)	3.33	0.000628
Chest	76 (9.06%)	4.79	763 (90.94%)	3.27	0.000013
Abdomen and pelvis	60 (7.15%)	4.67	779 (92.85%)	3.31	0.000427
Upper limb	228 (27.18%)	4.19	611 (72.82%)	3.14	0.000002
Lower limb	177 (21.1%)	4.43	662 (78.9%)	3.15	0.000000

**Table 5:** Distribution of patients with isolated injuries and their mean pain scores.

Part of Injury	Patients with Isolated Injury	Mean Pain Score	Standard Deviation
Head	385 (59.69%)	2.54	2.34
Neck	10 (1.55%)	3.40	3.20
Chest	24 (3.72%)	3.88	2.25
Abdomen and pelvis	23 (3.57%)	4.22	2.07
Upper limb	121 (18.76%)	4.16	2.96
Lower limb	82 (12.71%)	5.05	2.77
Total	645		

**Table 6:** Distribution of patients with or without treatment with their pain assessment.

Patients	With Assessed Pain (1–10)	Without Pain Assessed as “No Pain” (0)	Without Pain Assessment	Total
Treated with analgesics	183	2	23	208
Without analgesic treatment	468	186	119	773
Total	651	188	142	981

**Table 7:** Frequency of analgesic administration, pre-treatment mean pain scores reported by assessed patients, and mean pain score reduction after reassessment.

Analgesic Drug	Number of Treated Patients	Mean Pain Score	Standard Deviation (PS)	Number of Patients with Reassessed Pain	Mean Pain Reduction After Reassessment	Standard Deviation (PR)
Morphine alone	46	7.41	1.47	45	4.37	1.9
Paracetamol alone	43	5.35	2.28	40	2.08	1.99
Fentanyl alone	40	6.98	2.14	38	4.29	2.44
Morphine with paracetamol	14	7.14	1.83	14	3.79	2.67
Metamizole alone	9	5.22	1.92	8	1.88	1.25
NSAID alone	7	4.28	2.21	7	1.57	1.99
Paracetamol with metamizole	5	5.8	1.79	5	2.6	0.89
Fentanyl with paracetamol	5	9.2	1.3	5	5.2	2.17
Fentanyl with morphine	4	8	1.63	4	5.25	3.77
Fentanyl with metamizole	3	9.33	0.57	3	6.66	3.51
Morphine with metamizole	2	7	1.41	2	4	1.41
Paracetamol with NSAID	1	8	0	1	6	0
Morphine with paracetamol and metamizole	1	8	0	1	4	0
Morphine with NSAID	1	7	0	1	1	0
No treatment	657	2.5	2.2	565	0.41	1.03

**Table 8:** Distribution of non-pharmacological interventions administered to patients transported to the ED by EMS.

Non-Pharmacological Treatment	Number of Treated Patients	Frequency Among Non-Pharmacological Treatment
Dressing	266	63.94%
Immobilization	140	33.65%
Cervical collar	53	12.74%
Long spinal board	41	9.86%

**Table 9:** Distribution of reassessment time.

Reassessment Time in Minutes	Number of Reassessed Patients
<1	15
1–2	96
2–5	243
6–10	161
11–20	113
21–30	62
>31	44
Total	734

## Data Availability

The data presented in this study are available on request from the corresponding author.

## Abbreviations

The following abbreviations are used in this manuscript:

PMOH	Polish Ministry of Health
NSAID	Nonsteroidal anti-inflammatory drug
EMS	Emergency Medical Services
GCS	Glasgow Coma Scale
ED	Emergency Department
VAS	Visual Analog Scale
NRS	Numerical Rating Scale
FACE	Wong–Baker Faces Pain Rating Score
